# Supplementary material for: Perception, Trust, and Motivation in Consumer Behavior for Organic Food Acquisition: An Exploratory Study
Source: Foods. 2025 Jan 17;14(2):293. doi: 10.3390/foods14020293 (PMC11765215; doi:10.3390/foods14020293)
Supplement: Supplementary file 1 [file foods-14-00293-s001.zip › Figures S1 and S2 SM.pdf]

The winners of the edition 2024 of the EU Organic Awards are:

- **Best organic farmer (female)** to Ms Reinhilde Frech-Emmelmann in Austria. Ms Frech-Emmelmann founded ReinSaat GmbH in 1998 at a biodynamic Demeter farm in St. Leonhard am Hornerwald, Lower Austria. The farm specialises in organic, GMO-free seeds, with over 800 seed-resistant varieties, promoting biodiversity and sustainable farming across Europe (Figure S2, A-C).
- **Best organic farmer (male)** to Mr Benny Schöpf in Germany. Mr Schöpf is the chief vegetable grower at Kartoffelkombinat, the largest community-supported cooperative farm in Germany. Supplying 2,300 households with organic vegetables weekly, the farm prioritizes fair working conditions and sustainable practices, promoting an alternative agricultural economic system (Figure S2, D-F).
- **Best organic region** to South Savo in Finland. South Savo has built a strong organic farming culture through 40 years of collaboration between farmers, researchers, and local authorities. With 200 organic farms, the region promotes sustainable practices, preserving water quality and biodiversity, and is home to the Finnish Organic Research Institute.
- **Best organic city** to BioStadt Bremen in Germany. With over 30% of farms certified organic, the city promotes sustainable food systems through community projects and innovative farming initiatives, empowering citizens to drive local change. BioStadt Bremen is working towards converting all municipal catering in schools, crèches, and hospitals to 100% organic by 2025.
- **Best organic bio-district** to Sörmland Bio-district in Sweden. Located south of Stockholm, Sörmland has been a pioneer in organic farming since the 1940s, bringing together farms, food processors, restaurants, and more. With 20% of its farmland organic, the district promotes local organic products, sustainable tourism, and awareness of organic food's health benefits.
- **Best organic food processing SME** to Gino Girolomoni Cooperativa Agricola in Italy. Located in the Marche region, this cooperative specialises in organic pasta production, continuing the mission of its founder, Gino Girolomoni. With 80 hectares of organic farmland and renewable energy-powered facilities, it produces 9 million tons of pasta annually, supporting over 300 farmers and 60 local workers (Figure S2, G-I).
- **Best organic food retaile**r to SAiFRESC in Spain. Founded by three farmers in 2011, SAiFRESC transitioned to organic farming, revitalising agriculture in the Huerta de Valencia. With 30 hectares of organic land, they produce 70 organic products, selling 90% of their harvest locally and reducing packaging. The initiative promotes a circular economy and provides educational workshops on organic farming (Figure S2, J-L).
- **Best organic restaurant/food service** to a Kalf & Hansen in Sweden. Founded in 2014 by Rune and Fabian Kalf-Hansen, this restaurant chain offers 100% organic, seasonal Nordic cuisine. With two restaurants, catering services, and organic meals on Swedish trains, Kalf & Hansen prioritise local sourcing, sustainability, and affordable organic meals, building strong relationships with local producers (Figure S2, M-O).

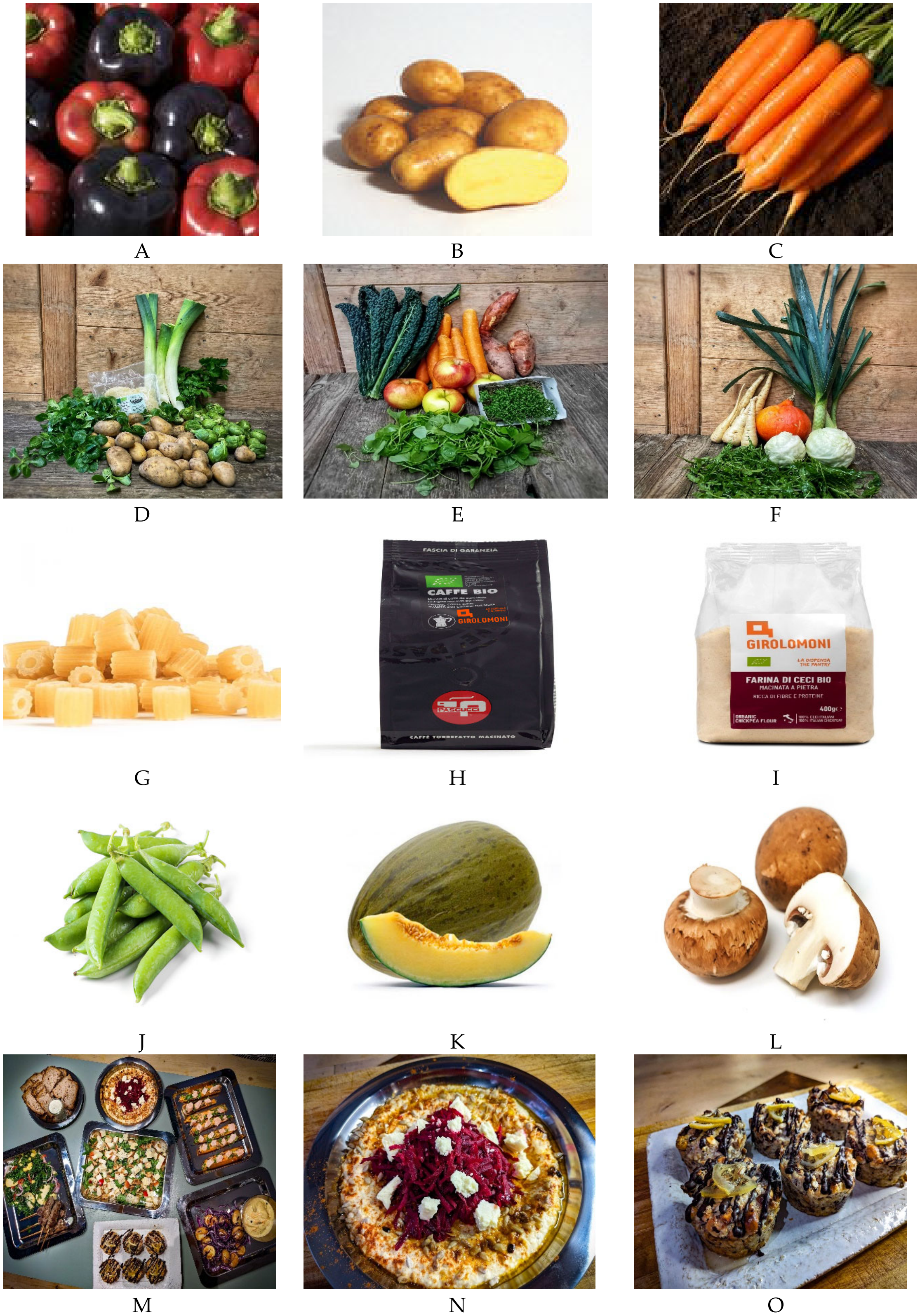

Figure S1. Organic foods produced by the winners of 2024 of the EU Organic Awards**: A-C** https://www.reinsaat.at/DE/; **D-F** https://www.kartoffelkombinat.de/ **G-H** https://girolomoni.it/ **J-L** https://saifresc.es/ **M-O** https://www.kalfochhansen.se/

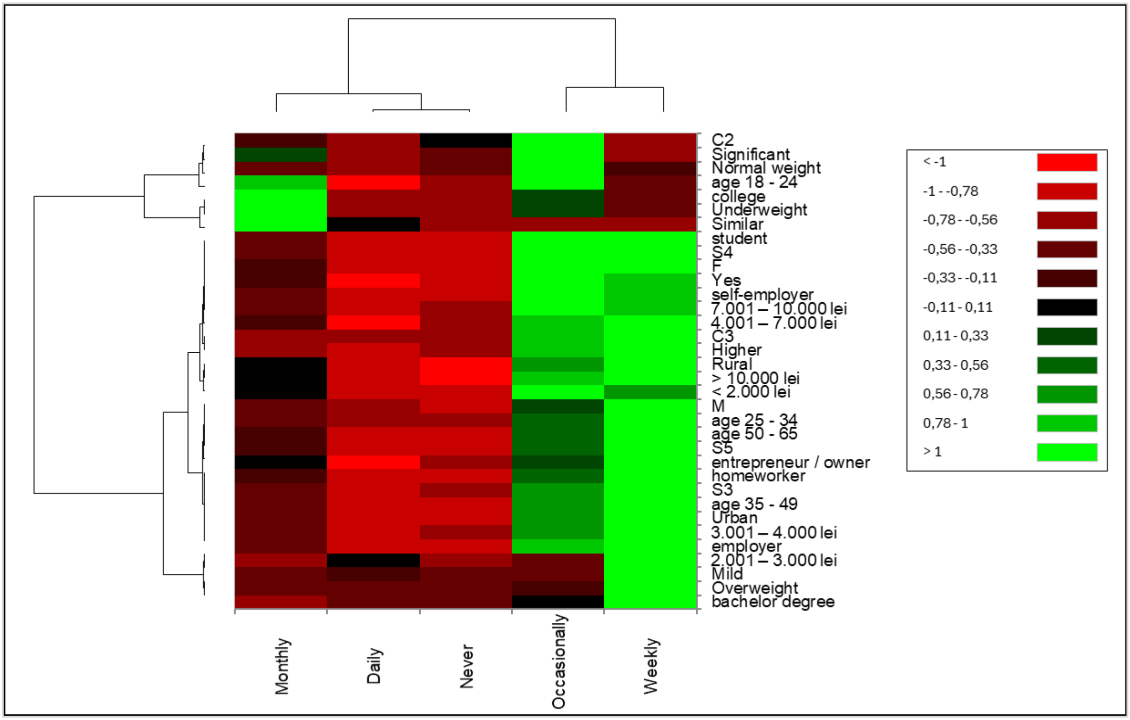
C2
Significant
Normal weight
age 18 - 24
college
Underweight
Similar
student
S4
F
Yes
self-employer
7.001 – 10.000 lei
4.001 – 7.000 lei
C3
Higher
Rural
> 10.000 lei
< 2.000 lei
M
age 25 - 34
age 50 - 65
S5
entrepreneur / owner
homeworker
S3
age 35 - 49
Urban
3.001 – 4.000 lei
employer
2.001 – 3.000 lei
Mild
Overweight
bachelor degree
Monthly
Daily
Never
Occasionally
Weekly
< -1
-1 - -0,78
-0,78 - -0,56
-0,56 - -0,33
-0,33 - -0,11
-0,11 - 0,11
0,11 - 0,33
0,33 - 0,56
0,56 - 0,78
0,78 - 1
> 1

A

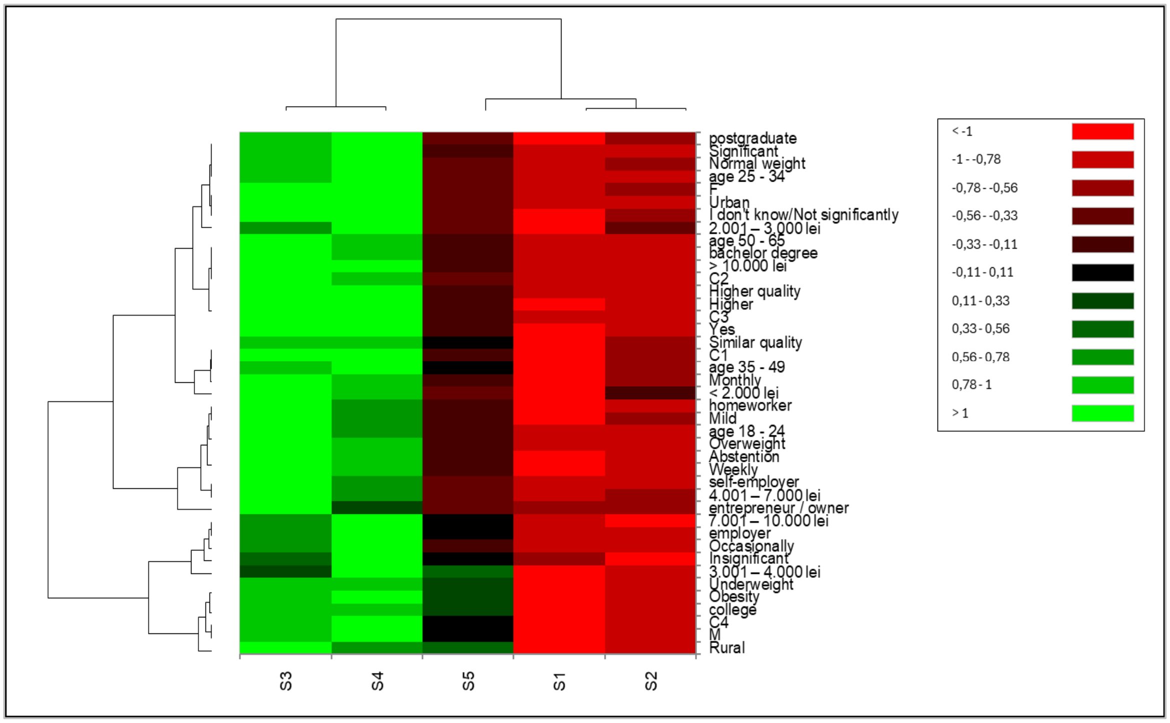
postgraduate
Significant
Normal weight
age 25 - 34
F
Urban
I don't know/Not significantly
2.001 – 3.000 lei
age 50 - 65
bachelor degree
> 10.000 lei
C2
Higher quality
Higher
C3
Yes
Similar quality
C1
age 35 - 49
Monthly
< 2.000 lei
homeworker
Mild
age 18 - 24
Overweight
Abstention
Weekly
self-employer
4.001 – 7.000 lei
entrepreneur / owner
7.001 – 10.000 lei
employer
Occasionally
Insignificant
3.001 – 4.000 lei
Underweight
Obesity
college
C4
M
Rural
S3
S4
S5
S1
S2
< -1
-1 - -0,78
-0,78 - -0,56
-0,56 - -0,33
-0,33 - -0,11
-0,11 - 0,11
0,11 - 0,33
0,33 - 0,56
0,56 - 0,78
0,78 - 1
> 1

B

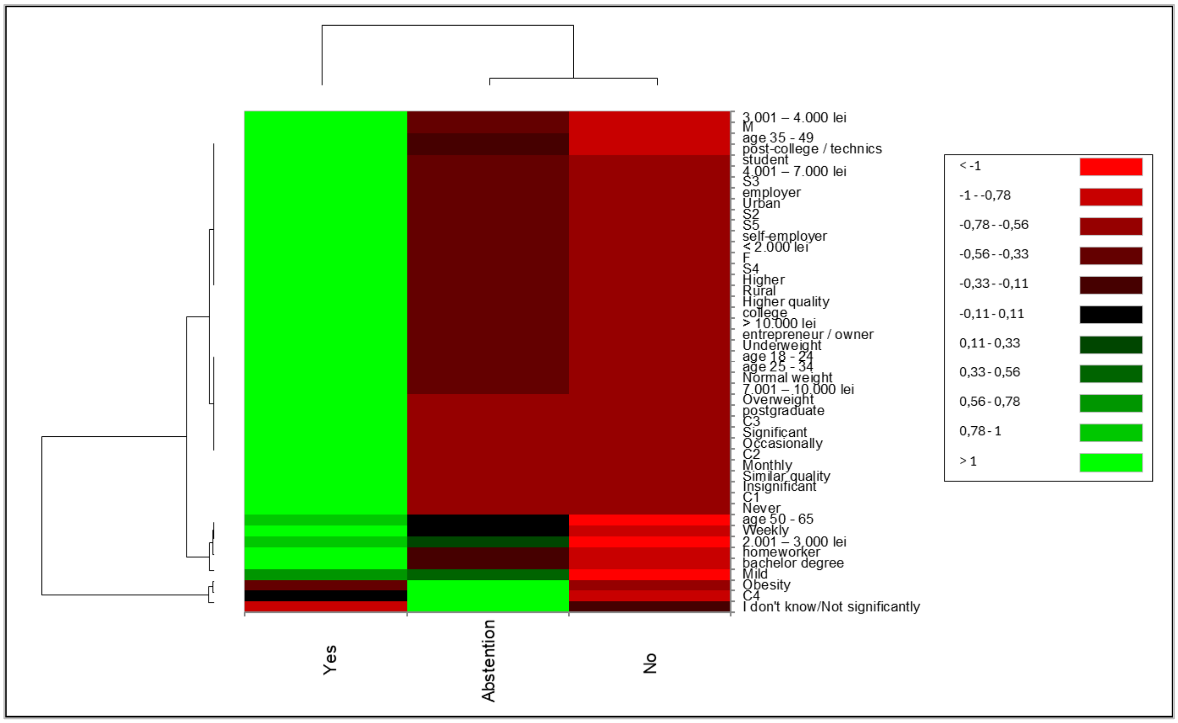

C

Figure S2. The most significant differences between all parameters involved in Food acquisition frequency (**A**), Eco-food satisfaction level S1-S5 (S1 – minimal, S5 – maximal) (**B**), Eco-food recommendation potential (**C**).

Nearly 100 applications were received from across the EU for this year's edition, with 24 candidates shortlisted from 11 countries. The EU Organic Awards feature 7 categories and 8 individual awards, recognising innovative, sustainable, and inspiring projects that add significant value to organic production and consumption. The awards are organised by the European Commission, the European Economic and Social Committee, the European Committee of the Regions, COPA-COGECA, and IFOAM Organics Europe, with support from the European Parliament and the Council, https://agriculture.ec.europa.eu/news/eight-winners-announced-third-eu-organic-awards-2024-09-23_en .
