# Supplementary material for: Perception, Trust, and Motivation in Consumer Behavior for Organic Food Acquisition: An Exploratory Study
Source: Foods. 2025 Jan 17;14(2):293. doi: 10.3390/foods14020293 (PMC11765215; doi:10.3390/foods14020293)
Supplement: Supplementary file 1 [file foods-14-00293-s001.zip › Online questionnaire.pdf]

This questionnaire is part of an interdisciplinary and the responses are used strictly for scientific purposes. Participation is voluntary, the answers will be completely anonymous, in accordance with the European ethical recommendations regarding the confidentiality of the data collected in this survey, as well as the anonymity and security of the participants (EU Regulation 2016/679 on the protection of individuals with regard to the processing of personal data). By completing this form, the participants agree to the anonymous processing of personal data.

I. Demographics

1. Please select your sex:

A. Female

B. Male

2. Please select the age:

A. 18 years

B. 19-24 years

C. 25-34 years

D. 35-49 years

E. 50-65 years

F. > 65 years

3. Please specify your residence:

A. Rural

B. Urban

4. Please specify your highest degree:

A. bachelor's degree

B. college

C. high school

D. middle

E. post-college / technical

F. postgraduate

5. Please specify your occupation:

A. employer

B. entrepreneur / owner

C. homeworker

D. pensioner

E. self-employer

F. student

G. unemployed

6. Please specify your monthly income:

A. RON 2001 – 3000

B. RON 3001 – 4000

C. RON 4001 – 7000

D. RON 7001 – 10,000

E. RON <2000

F. RON >10,000

7. Please mention your high (m)

8. Please mention your weight (kg)

Based on data from questions 7 and 8 we calculated BMI and classified the respondents in 4 categories:

A. Underweight

B. Normal weight
C. Overweight
D. Obese

II. Eco-food concept

9. How do you understand about eco-food?

A. Food from rural households

B. Food with UE ECO sign

C. Food with BIO/ECO/Organic mention on the package

D. Food with Natural/100% Natural mention on the package

10. Do you think that organic food are healthier than conventional ones?

A. Yes

B. Maybe

C. No

D. I do not know/ No significant difference

11. How do you perceive the eco-food quality compared to conventional ones?

A. Higher quality

B. Lower quality

C. Similar quality

D. I do not know/No significant difference

12. Please mention the most significant aspects correlated with organic foods:

A. Limits for pesticides and additives

B. Higher nutritional value

C. Food safety

D. Sustainability and environmental impact

13. Do you think that eco-food has more benefits on environment than conventional ones?

A. Yes

B. Maybe yes

C. Maybe no

D. No

E. I don’t know

14. Do you think that eco-food has more benefits on your health than conventional ones?

A. Yes, significant

B. No

C. Not significant

15. How do you perceive the eco-food prices compared to conventional ones?

A. Higher

B. Lower

C. Similar

D. No significant difference

16. Please mention the source of first information about eco-food:

A. Advertising

B. Expo

C. Family/friends

D. Internet

E. Supermarket

F. TV

17. Do you think that the currently available information about eco-food is enough?

A. Enough

B. Moderate

C. Not enough

D. I don't know

18. How is your updating rate about eco-food production and provenance?

A. Regularly

B. Frequently

C. Occasionally

D. Rarely

E. Never

19. Please mention the rate of organic-food verifying during acquisition:

A. Always

B. Never

C. Occasionally

D. Rarely

20. Please specify the rate of eco-food ingredients verifying from the labels:

A. Always

B. Never

C. Occasionally

D. Rarely

21. Please appreciate your confidence level in the eco-food label:

A. C1 (the lowest level)

B. C2

C. C3

4. C4

5. C5 (the highest level)

22. Please mention if you have confidence in eco-food certified in Romania:

A. Yes

B. No

C. I don't know

III. Eco-food acquisition behavior

23. Please specify the frequency of eco-food acquisition:

A. Daily

B. Weekly

C. Monthly

D. Occasionally

E. Never

24. Please indicate the preferred organic foods:

A. Fruits and vegetables

B. Dairy products and eggs

C. Fish and meat

D. Honey and other healthy food

E. Basic foods

F. Sweets

G. Novel foods

25. Please indicate the place of eco-food acquisition:

A. Supermarket/hypermarket

B. Market

C. Pharmacy or healthy food store

D. Online

E. Neighborhood store

F. Other

26. Please mention the main criteria for organic food acquisition:

A. Eco-friendly package

B. Aspect

C. Taste and odor

E. Friend/family recommendations

F. Provider and country

G. Price

27. Please mention the main reason for organic food acquisition:

A. Higher quality

B. Personal healthcare priority

C. Environmental care

D. Limits for pesticides and additives

E. Support for local farmers

28. Please mention the main reason to avoid organic foods:

A. High price

B. No confidence

C. Missing data about benefits

D. Low availability

29. Please show the satisfaction level of organic food consumption:

A. S1 (the lowest level)

B. S2

C. S3

D. S4

E. S5 (the highest level)

30. Do you recommend organic food consumption?

A. Yes

B. No

C. Abstention
